# Café Food Safety and Its Impacts on Intention to Reuse and Switch Cafés during the COVID-19 Pandemic: The Case of Starbucks

**DOI:** 10.3390/ijerph20032625

**Published:** 2023-02-01

**Authors:** Yunho Ji, Won Seok Lee, Joonho Moon

**Affiliations:** 1 Department of Tourism Administration, Kangwon National University, Chuncheon 24341, Republic of Korea; 2 Department of Tourism and Recreation, Kyonggi University, Seoul 03746, Republic of Korea

**Keywords:** food safety, intention to revisit, freshness, quarantine, hygiene, healthiness

## Abstract

We explored the definition of food safety in the coffee service business during the 2019 coronavirus disease (COVID-19) pandemic because consumer values and decision-making may have been affected by the pandemic. The food safety dimensions are freshness, quarantine, hygiene, and healthiness. We evaluated the effects of café food safety on both the consumer intention to revisit a café and their intention to switch to other cafés. We used the Amazon Mechanical Turk system for data collection. In total, 474 individuals responded to the survey questions. We used the statistical package for the social sciences (SPSS) ver. 20.0 and the analysis of moment structure (AMOS) ver. 21.0. We subjected the definition of café food safety to confirmatory factor analysis and then used structural equations to test the research hypotheses. The four dimensions adequately defined food safety. The results indicated that food safety positively influenced the intention to revisit, although it had no significant impact on the intention to switch cafés. Our findings will assist managers because we identify the implications of food safety for the coffee service business.

## 1. Introduction

Businesses must maintain friendly relationships with customer stakeholders; this is a key element of corporate social responsibility [1,2,3]. Bad food makes people ill; food safety scandals substantially damage businesses [4,5,6,7]. The coffee service sector must carefully consider these points; café business grew globally from USD 192 billion in 2020 to USD 244 billion in 2021. Safe food is imperative. Here, we define food safety in the café context and explore the impact of safe food on business. There is a vast body of literature regarding food safety [8,9,10,11], clean food [8,12,13,14], fresh food [9,15], and healthy food [16,17]. Such foods enhance consumer health and minimize risk. The 2019 coronavirus disease (COVID-19) pandemic caused a substantial enhancement of consumer interest in food safety [18,19]. Customers were at a high risk of disease transmission when eating with others in public spaces [20,21]. Thus, we explored quarantine as an unusual dimension of café food safety during the pandemic. Despite the importance of café food safety, there has been minimal research on what constitutes such safety or what beliefs are held by consumers; the present study explored these aspects.

The outcome variable was an intention to revisit a café/switch to a new café (i.e., opposite consumer intentions). An intention to revisit retains customers [22,23,24], whereas an intention to switch loses them [25,26,27]. We selected Starbucks as the research subject because the organization holds the largest single market share (approximately 40% as of 2019) of the café business [28] associated with readily available public data. Additionally, the popularity of the chain may encourage responders to relate individual detailed experiences. The present study adds to the literature on food safety in the café sector and provides information that café business managers will find useful.

## 2. Review of the Literature and Research Hypotheses

### 2.1. Food Safety and Corporate Social Responsibility

Food safety is a food-handling protocol that prevents consumer illness (perceived by consumers as a risk) [11,29,30]. Safe food promotes health and reduces the risk of cardiovascular disease [31,32,33]. Successful businesses both serve safe food and emphasize its safety to consumers [33,34,35]. Numerous studies have emphasized the importance of incorporating food safety into corporate social responsibility; this protects the principal stakeholders (i.e., consumers) [11,36,37]. The provision of safe food builds business sustainability, ensuring that customers often revisit; food scandals substantially damage business reputations [29,37,38]. Safe food is becoming increasingly imperative; customers prioritize both their short- and long-term health [30,36,38]. Indeed, consumers tend to pay more for food they believe to be safe, encouraging businesses to focus on a safe food supply [11,36,39]. Corporate wrongdoing (poor food preparation) leads to penalties; businesses lose market share and reputation, resulting in decreased share prices [4,5,6,7]. Food businesses must maintain good relationships with customers [30,37].

The first dimension of food safety is freshness (i.e., consumer assessment of whether food is properly cooked from raw ingredients); freshness is linked to sensory appeal [8,40,41]. Freitas et al. [42] found that such appeal encouraged consumers to feel that fresh food is safe. Rotten food is completely unacceptable [14,43]. Food freshness, which protects health, is the most basic aspect of food service because the associated sensory appeal controls customer impressions [13,14,44].

Quarantine is the second dimension of food safety; workers protect consumers from infections by wearing masks, frequently using hand sanitizer [45,46], and checking customer temperatures and vaccination cards [27,47]. During the pandemic, these practices were considered essential [48,49,50]. Specifically, consumers favored businesses with strict quarantine guidelines that protected customers from infection [45,51,52]. Customers eschewed eating in crowded places and relied heavily on food delivery systems [53].

The third dimension of food safety is hygiene. Poor sanitation increases the risk of foodborne illnesses such as fever, nausea, and dehydration [54,55,56]. Good hygiene protects food against chemical and biological contamination during production [56,57]. Good hygiene protects consumers [58,59] by decreasing the risk of contamination [9,12,55].

The fourth dimension of food safety is healthiness. Bad food promotes obesity [60], diabetes [16,17], high blood pressure [16], and heart conditions [16,60]. Indeed, bad food can adversely affect mental health [60,61,62,63]. The market exhibits increased interest in healthy food, and harmful items are removed during production; genetically modified materials and undesirable organic products are carefully monitored and traced, allowing the information to be shared with consumers [64,65].

### 2.2. Switching and Revisiting Intentions

In the marketing literature, switching intention refers to the likelihood that consumers will buy competitors’ products or services [26,66,67]. Consumer switching decreases sales and thus market share [25,27,68]. Many efforts have been made to understand and prevent switching. Shin and Kim [27] and Peng et al. [68] explored the attributes of switching intentions in the context of mobile services. Han et al. [26] defined the determinants of switching intention in the lodging sector. Liang et al. [67] evaluated the antecedents of switching intention among Airbnb users. Jung, Han, and Oh [69] examined airline customer behaviors, using switching intention as a dependent variable. Similarly, Nikbin, Marimuthu, and Hyun [70], as well as Kim [71], used switching intention as an explained variable in the context of restaurant business. An intention to reuse (or revisit) refers to the likelihood that consumers will repeat purchases of a specific company’s goods or services [23,72,73]. Intention to reuse is a form of loyalty that improves sales, market share, and business sustainability [22,24,74]. Many studies have regarded intention to reuse as a principal attribute. For instance, Son, Bae, and Lee [75] used intention to reuse as a dependent variable when assessing service agility. Li et al. [73] studied intention to reuse in the educational context, and Park [76] examined intention to reuse among airline customers. Bao and Zhu [24] used intention to reuse as a principal element when evaluating the behaviors of individuals who ordered food deliveries.

Several studies have provided empirical evidence of the relationship between consumer intention and food safety. For instance, a meta-analysis by Lin and Roberts [77] showed that food safety was significantly associated with consumer behavioral intentions. Wang and Tsai [11] reported that food safety was an essential antecedent of customer loyalty. Lee, Tsai, and Ruangkanjanases [78] revealed that food safety positively affected an intention to repurchase; Seo and Lee [10] found the same result in a study of street food consumers. Shim et al. [27] identified food safety as an essential consideration in terms of customer reuse intention in the café domain. Zhang, Ma, and Morse [37] and Wang et al. [79] empirically demonstrated that corporate social responsibility in terms of food safety positively affected consumer decision-making. Nguyen, Yeh, and Huang [80] found that a perceived risk of food contamination caused consumers to switch. Given these data, we proposed the following research hypotheses:

**Hypothesis** **1_0_ (H1_0_).** *Food safety does not significantly affect switching intention among café customers.*

**Hypothesis** **1_a_ (H1_a_).** *Perceived poor food safety increases switching intention among café customers.*

**Hypothesis** **2_0_ (H2_0_).** *Food safety does not significantly affect revisiting intention among café customers.*

**Hypothesis** **2_a_ (H2_a_).** *Perceived good food safety increases revisiting intention among café customers.*

## 3. Methods

### 3.1. Research Model and Data Collection

**Figure 1:**
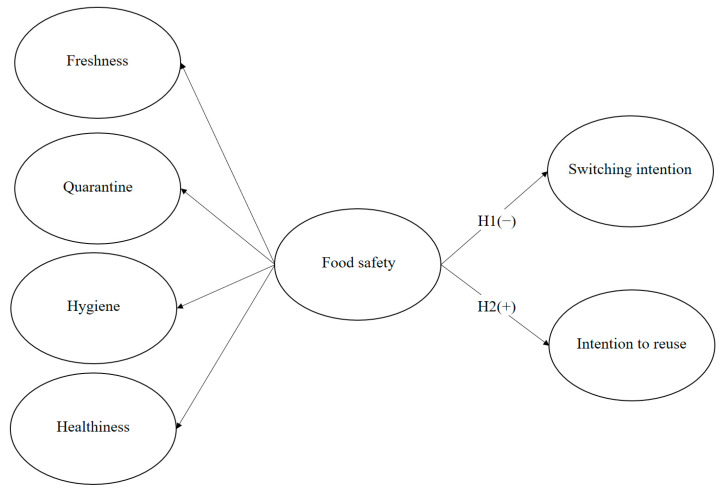
Research model.

Figure 1 illustrates the research model. The dimensions of food safety are freshness, quarantine, hygiene, and healthiness. These serve as secondary elements when testing the structural relationships among food safety, switching intention, and intention to revisit. Food safety (a second-order factor) was hypothesized to positively affect the intention to revisit and to negatively affect switching intention.

We used Amazon Mechanical Turk to recruit survey participants (http://www.mturk.com, accessed 7 December 2020). This method has been widely adopted by researchers of consumer behaviors, and many studies have demonstrated statistically significant outcomes [81,82,83,84]. Data were collected on 7 and 10 December 2020; we received 474 valid observations. The geographical area was constrained to the US. All respondents were familiar with Starbucks, which is popular in the US. Amazon Mechanical Turk adequately collects survey participants from the US.

### 3.2. Measurement Items

All measurement items were derived from the literature after a modification to match the aims of the current work, and all respondents rated the items on 5-point Likert scales (1 = strongly disagree, 5 = strongly agree). Freshness is the consumer rating of food taste and the ingredients used [14,41,44]. Quarantine refers to how well a business followed COVID-19 containment measures from the consumer perspective [21,47,49]; hygiene refers to operations and food preparation cleanliness from the consumer perspective [9,55,85,86]; and healthiness refers to health-promoting aspects of café food [16,17,87]. Switching intention refers to the consumer likelihood of switching to a different vendor for future purchases [25,26,68]. Intention to reuse indicates the likelihood that a consumer will use a specific brand or product again [24,73,75]. Table 1 presents the measurement items. With the exception of switching intention (three items), all constructs included four items. The derived constructs were freshness, quarantine, hygiene, healthiness, switching intention, and intention to reuse.

### 3.3. Data Analysis

We first performed a frequency analysis of respondent demographic characteristics. Confirmatory factor analysis was used to check convergent validity. As indicated in the literature, the convergent validities of measurement items were ensured by using multiple criteria (loading > 0.5, average value extracted [AVE] > 0.5, and construct reliability < 0.7) during confirmatory factor analysis [88,89,90]. We derived univariate statistics and engaged in structural equation modeling. Univariate statistics were used to provide general descriptions. We used the four dimensions of food safety (freshness, quarantine, healthiness, and hygiene) as second-order factors. We computed the squares of the correlation coefficients and then compared these values with the AVE. This approach ensured discriminant validity when the AVE was larger than the squared correlation coefficient.

## 4. Results

### 4.1. Demographic Information

Table 2 presents the profiles of the survey participants: 289 men and 185 women. Age analysis revealed that 78% of the participants were in their 20s or younger or in their 30s (369 of 474). In terms of monthly household income, the largest proportion (138 of 474) earned USD 2000–3999. In terms of visit frequencies, most (192 of 474) visited Starbucks once or twice per week (40.5%).

### 4.2. Confirmatory Factor Analysis and Correlation Matrix

Table 3 shows the results of confirmatory factor analysis. The goodness-of-fit indices indicate that the results are statistically significant. All factor loadings are greater than the threshold. Both the construct reliability and the AVE satisfied the criteria for significance. Overall, the convergent validities of measurement items were confirmed. Table 3 lists the means and standard deviations of the measured items. Among the four food safety dimensions, healthiness was rated lowest and freshness was rated highest. The mean values of switching intention and intention to reuse indicated that most Starbucks consumers planned to remain rather than leave.

Table 4 shows the correlation matrix. Food safety was positively correlated with the intention to revisit (r = 0.846, *p* < 0.05). Switching intention was negatively correlated with the intention to revisit (r = −0.158, *p* < 0.05). However, food safety was not significantly correlated with switching intention. Moreover, the squared correlation coefficients were less than the AVE, confirming the discriminant validities of the principal attributes explored.

### 4.3. Hypothesis Testing

Table 5 illustrates the results of hypothesis testing. The results of the structural equation model were statistically acceptable according to the goodness-of-fit indices. Food safety exerted a positive effect on the intention to revisit (β = 0.848, *p* < 0.05), but it had no significant impact on switching intention. Therefore, only H1 was supported by the model.

## 5. Discussion

We examined four dimensions of food safety (a corporate social responsibility activity) at Starbucks, as a representative of the coffee service industry. To ensure validity, we performed a confirmatory factor analysis; four attributes of food safety (freshness, quarantine, hygiene, and healthiness) were identified as relevant to cafés. In terms of means, consumers highly rated coffee freshness and hygiene. Consumers consider Starbucks products to be fresh; they presume that Starbucks food preparation and its beverage preparation are sanitary. However, the quarantine and healthiness ratings were slightly less positive. Customers may have perceived a small problem with quarantine; this was presumably because other customers did not follow guidelines in public places, although the employees wore masks and checked temperatures, and ventilation was good. Additionally, coffee consumption is controversial in terms of healthiness. In small amounts, coffee promotes health, but excessive caffeine and sugar must be avoided. The descriptive statistics indicated a moderately strong switching intention, along with a stronger intention to revisit. Café food safety positively affected the intention to revisit. Shim et al. [21] found a significant association between safe food and café loyalty. However, we found only a nonsignificant association between food safety and switching intention. Food safety did not appear to influence whether consumers may choose a different café. Unlike Nguyen et al. [80], who found a significant effect of food safety on switching intention for agricultural products, we found only a nonsignificant association between food safety and switching intention, possibly because cafés are very competitive and because consumers have many options. Thus, an emphasis on food safety alone may not be sufficient to persuade consumers to stay. In the café sector, it may be difficult (and costly) to retain all customers.

## 6. Conclusions

### 6.1. Theoretical and Practical Implications

This study contributes to the literature by refining the definition of food safety in the context of a café. We identified four dimensions of food safety and confirmed their relevance by confirmatory factor analysis. We provided insights into the relationship between food safety and customer loyalty (i.e., the intention to revisit). Food safety exerted a substantial impact on the intention to revisit, confirming the external validity of our current research compared with the approaches in previous studies [10,78]. However, food safety did not significantly reduce switching intention, possibly because there are many cafés among which consumers can choose. By revealing this distinctive coffee-consumer characteristic, the present study contributes to the literature on the intention to revisit and switch.

This study has practical implications. First, café managers must foster safety-related attributes to increase the numbers of customers. Fresher products and ingredients in terms of both coffees and side menus are necessary. Café managers also need to offer the freshness of information to customers by revealing roasting date and producing date. For some time, mask wearing by both employees and customers, as well as temperature checks and free hand sanitizer, must continue. Kitchens must be very clean; the visibility of the kitchen needs to be elevated because it could work as physical evidence for customers to evaluate food safety. Plus, offerings high in sugar and caffeine should be optional for customers with the increasing awareness of choices regarding caffeine and sugar levels. Customer revisits would then increase, leading to greater sales and a larger market share. Furthermore, café managers should allocate more resources to quarantine and food healthiness; consumers rated these areas slightly lower than freshness and hygiene.

### 6.2. Suggestions for Future Research

Our work had some limitations. First, we sampled only café customers; there are other food service sectors. The use of additional dependent variables may confirm the influence of food safety. Second, the food safety definition may be refined in the future to better reflect market characteristics. Such efforts would increase the broader understanding of café customers.

## Figures and Tables

**Table 1:** Measurement items.

Construct	Code	Item
Freshness	FR1	The taste of Starbucks products is great.
	FR2	Starbucks offers fresh products.
	FR3	Starbucks products are delicious.
	FR4	Starbucks uses fresh ingredients.
Quarantine	QR1	Starbucks is good at COVID-19 quarantine.
	QR2	Starbucks adequately maintains COVID-19 quarantine.
	QR3	Starbucks employees perform well in terms of COVID-19 quarantine.
	QR4	COVID-19 quarantine is effectively implemented at Starbucks.
Hygiene	HY1	Starbucks products are hygienic.
	HY2	Starbucks products are clean.
	HY3	Sanitation of Starbucks goods is effectively managed.
	HY4	Starbucks food is clean and hygienic.
Healthiness	HE1	Starbucks products are healthy.
	HE2	Starbucks products improve my health.
	HE3	Starbucks offers products that are not good for health.
	HE4	Starbucks products are low in calories.
Switching intention	SI1	I will use other goods instead of Starbucks goods.
	SI2	I will buy another brand of coffee, rather than Starbucks.
	SI3	I intend to switch from Starbucks products.
Intention to reuse	IR1	I will reuse Starbucks.
	IR2	I will visit Starbucks again.
	IR3	I intend to purchase Starbucks products again.
	IR4	I am willing to repurchase Starbucks products.

**Table 2:** Profile of survey participants (*n* = 474).

Item	Frequency	Percentage
Male	289	61.0
Female	185	39.0
Age: 20s or younger	188	39.6
30s	181	38.2
40s	60	12.7
50s or older	45	9.5
Monthly household income		
<USD 2000	94	19.8
USD 2000–3999	138	29.1
USD 4000–5999	97	20.5
USD 6000–7999	45	9.5
USD 8000–9999	47	9.9
>USD 10,000	53	11.2
Weekly visiting frequency		
<1 time	163	34.4
1–2 times	192	40.5
3–5 times	94	19.8
>5 times	25	5.3

**Table 3:** Measurement items.

Construct (AVE)	Subdimension	Code	Mean	SD	Loading	CR
Food safety (0.582)	Freshness	FR1	4.11	0.89	0.710	0.939
		FR2	3.93	0.89	0.723	
		FR3	4.13	0.86	0.718	
		FR4	3.87	0.90	0.721	
	Quarantine	QR1	3.75	0.97	0.804	
		QR2	3.76	0.94	0.836	
		QR3	3.89	0.88	0.759	
		QR4	3.82	0.88	0.780	
	Hygiene	HY1	4.04	0.85	0.789	
		HY2	4.05	0.87	0.780	
		HY3	4.02	0.87	0.763	
		HY4	4.03	0.88	0.773	
	Healthiness	HE1	3.32	1.17	0.859	
		HE2	3.14	1.23	0.861	
		HE3	3.33	1.11	0.752	
		HE4	3.18	1.21	0.806	
Switching intention (0.551)		SI1	3.33	1.15	0.769	0.783
		SI2	3.23	1.22	0.831	
		SI3	3.18	1.16	0.608	
Intention to reuse (0.685)		IR1	4.08	0.95	0.798	0.897
		IR2	4.16	0.91	0.828	
		IR3	4.16	0.92	0.848	
		IR4	4.08	0.95	0.836	

Note: AVE: average value extracted, CR: construct reliability, SD: standard deviation. χ^2^ = 860.698, df = 223, Q (χ^2^/df) = 3.860, goodness-of-fit index = 0.836, normed fit index = 0.875, relative fit index = 0.859, incremental fit index = 0.905, Tucker–Lewis index = 0.891, comparative fit index = 0.904, root-mean-square error of approximation = 0.078.

**Table 4:** Correlation matrix.

	1	2	3
**1. Food safety**	0.763		
**2. Switching intention**	0.067 (0.001)	0.742	
**3. Intention to reuse**	0.846 * (0.715)	–0.158 * (0.002)	0.828

Note: * *p* < 0.05. The diagonal is the square root of the AVE; values in parentheses are the squares of correlation coefficients.

**Table 5:** Hypothesis testing.

H	Path	Standard Beta	*p*-Value	Result
H1	Food safety→Switching intention	0.004	0.936	Not supported
H2	Food safety→Intention to reuse	0.848 *	0.000	Supported

Note: * *p* < 0.05, χ^2^ = 895.550, df = 224, Q (χ^2^/df) = 3.998, goodness-of-fit index = 0.828, normed fit index = 0.870, relative fit index = 0.854, incremental fit index = 0.900, Tucker–Lewis index = 0.886, comparative fit index = 0.899, root-mean-square error of approximation = 0.080.

## Data Availability

Not applicable.
